# Emergence of the rtA181T/sW172* mutant increased the risk of hepatoma occurrence in patients with lamivudine-resistant chronic hepatitis B

**DOI:** 10.1186/1471-2407-11-398

**Published:** 2011-09-21

**Authors:** Chau-Ting Yeh, Tiffany Chen, Chao-Wei Hsu, Yi-Cheng Chen, Ming-Wei Lai, Kung-Hao Liang, Tse-Ching Chen

**Affiliations:** 1 Liver Research Center, Department of Hepato-Gastroenterology, Chang Gung Memorial Hospital, Taipei, Taiwan; 2 Molecular Medicine Research Center, Chang Gung University, Taoyuan, Taiwan; 3 School of Medicine, New York University, New York, USA; 4 Department of Pediatric Gastroenterology, Chang Gung Children Hospital, Taoyuan, Taiwan; 5 Department of Pathology, Chang Gung Memorial Hospital, Taipei, Taiwan

## Abstract

**Background:**

Development of the hepatitis B virus (HBV) rtA181T/sW172* mutant could occur during prolonged lamivudine (LAM) therapy, conferring cross resistance to adefovir. Recent studies demonstrated an increased oncogenic potential of this mutant in NIH3T3 cells. In this study, we aimed to investigate the clinical significance of this finding.

**Methods:**

Serum samples from 123 LAM-resistant chronic hepatitis B patients were submitted for virological assays. A highly sensitive amplification created restriction enzyme site (ACRES) method was devised to detect small amounts of the rtA181T mutant in the serum. Virological factors including HBV-DNA level, genotype, precore G1896A, BCP A1762T/G1764A, rtM204I/V, rtA181T and pre-S internal deletion mutations as well as clinical variables including subsequent use of rescue drugs were submitted for outcome analysis.

**Results:**

By use of the highly sensitive ACRES method, the rtA181T mutant was detectable in 10 of the 123 LAM-resistant patients. During the mean follow-up period of 26.2 ± 16.4 months (range 2 to 108 months), 3 of the 10 (30.0%) rtA181T-positive patients and 2 of the 113 (1.8%) rtA181T-negative patients developed hepatocellular carcinoma (HCC). Kaplan-Meier analysis indicated that the presence of rtA181T mutation (P < 0.001), age > 50 years (P = 0.001), and liver cirrhosis (P < 0.001) were significantly associated with subsequent occurrence of HCC. All 5 HCC patients belonged to the older age and cirrhosis groups.

**Conclusions:**

Emergence of the rtA181T/sW172* mutant in LAM-resistant patients increased the risk of HCC development in the subsequent courses of antiviral therapy.

## Background

In the past decade, much progress has been made in antiviral therapy for chronic hepatitis B virus (HBV) infection [1-3]. Several approved therapeutic agents are now available, including regular or pegylated interferon and nucleoside/nucleotide analogues (NA) such as lamivudine (LAM), adefovir dipivoxial (ADV), entecavir (ETV), telbivudine and tenofovir [3-7]. Although NAs are very effective in inhibiting HBV reverse transcriptase, long-term usage of NAs is required to reduce the risk of hepatitis relapses on drug withdrawal. This strategy leads to development of drug resistance, a major challenge for hepatologists. The risk of developing LAM resistance is 14-32% in the first year and up to 70% by the fifth year [4]. The most frequently encountered LAM-resistant mutant is rtM204V/I, which possesses a mutation located at the catalytic YMDD motif [8-11]. The rtL180M mutation usually occurs concurrently with the rtM204V mutation and serves as a compensatory mutation [11]. The rtA181T mutation has also been reported in a substantial proportion of LAM-resistant patients, which has been shown to confer LAM-resistance in cell-based assays [10]. Because of the overlap between the S and polymerase genes, a great proportion of patients carrying the rtA181T mutation also possessed the sW172* nonsense mutation, resulting in truncation of the pre-S/S reading frames [10].

The risk of developing ADV resistance was 2-3% and 28-29% by the second and fifth years of monotherapy in treatment naïve patients respectively [12-14]. The risk increased to 21% in LAM-resistant patients who were switched to ADV monotherapy after 1.5 years of ADV therapy [2,3]. The major ADV resistant mutants were rtN236T and rtA181T/V [5]. Resistance to ETV is rare for treatment naïve patients (1.5% by the fifth year) [2]. However, in the presence of rtM204I/V mutations, ETV resistance can occur if the rtI169T, rtT184A/F/G/I/L/S, rtS202G/I, or rtM250V mutation coexists [15,16]. The risk of developing telbivudine resistance was 2-3% and 21% after 1 and 2 years of therapy in treatment-naïve HBeAg-positive patients respectively. The major resistant mutant was rtM204I [17]. Tenofovir has recently been found to be effective in suppressing HBV replication with a low risk of drug resistance and several reports have shown that this drug is effective against various NA resistant or cross-resistant mutants [18,19].

It has been demonstrated that in patients with compensated cirrhosis, LAM therapy significantly reduces the risk of liver failure and hepatocellular carcinoma (HCC) [20]. However, in patients who developed LAM resistance, this beneficial effect was drastically compromised. Several strategies of therapy have been examined in LAM resistance patients, including withdrawal of LAM therapy, switching to ADV monotherapy, ADV add-on treatment, and switching to ETV monotherapy. The latter two strategies are accepted in several therapeutic guidelines [21]. Despite various efforts to develop effective rescue therapy for LAM-resistant patients, severe consequences such as HCC and liver failure still occur in a small number of patients [22-24].

Previous studies have indicated that several virological factors including HBV-DNA level, HBV e antigen (HBeAg) status, genotype, pre-S internal deletion mutant and basal core promoter (BCP) A1762T/G1764A mutation are closely associated with development of HCC in treatment naïve, chronic hepatitis B patients [25]. However, the associated virological factors for severe disease outcomes in patients with LAM-resistant chronic hepatitis B have not been clearly delineated. A major reason these patients are started on long-term LAM treatment early in the course of their disease is to prevent the occurrence of HCC. The development of LAM-resistance significantly compromises the effectiveness of this strategy. It is imperative that the clinical and virological factors associated with the development of HCC in this group of patients be delineated. In this study, we analyzed the clinical variables, virological parameters, and use of rescue agents to identify factors associated with severe disease outcomes.

## Methods

### Patients

This study was conducted under the approval of the institutional review board of Chang Gung Medical Center. The clinical records of 157 patients who received LAM and developed drug resistance from January 2002 to December 2007 were reviewed. Of these patients, 123 were included because they had serum samples stored in the Liver Research Center Serum Bank that could be retrieved for virological analysis. All patients were positive for hepatitis B surface antigen (HBsAg) but negative for antibody against hepatitis D virus or antibody against hepatitis C virus. All patients were also tested negative for human immunodeficiency virus infection. The following clinicopathological data were retrospectively reviewed: gender, age, presence of liver cirrhosis, HBeAg status, aspartate aminotransferase (AST), alanine aminotransferase (ALT), alpha-fetoprotein (AFP), the duration of LAM use before inclusion, subsequent use of ADV or ETV, total duration of NA use, and development of HCC or liver failure. None of the patients had detectable liver tumors (except hemangiomas) during the 3 years period prior to entry into this study. Monitoring of HCC occurrence was performed by measurement of AFP levels and ultrasonography every 3 months in cirrhotic patients and every 6 months in non-cirrhotic patients. Diagnosis of HCC was made by either echo-guided liver biopsy, fine needle aspiration cytology, high AFP level (> 200 ng/mL) plus at least one dynamic imaging study (dynamic computed tomography or magnetic resonance imaging), or one dynamic imaging study plus angiography (if alpha-fetoprotein < 200 ng/mL).

Serum hepatitis markers, including HBsAg, HBeAg, and antibody to hepatitis D virus were assayed by commercially available kits (Ausria-II and HBeAg-RIA, Abbott Laboratories, North Chicago, IL; and anti-HD; Formosa Biomedical Technology Corporation, Taiwan). Serum antibody to hepatitis C virus was assayed using a third-generation enzyme immunoassay kits (HCV EIA III; Abbott Laboratories).

### Virological assays

The HBV-DNA concentration was quantified by use of Roche COBAS AmpliPrep/COBAS Taqman HBV Test (Roche Diagnostics, Basel, Switzerland). The detection limit of this test was 69 copies/mL. In this test, 5.82 copies/mL was equivalent to 1 IU/mL. HBV genotypes were determined using the restriction fragment length polymorphism method or phylogenetic sequence analysis [26]. The methods to detect HBV basal core promoter (BCP) A1762T/G1764A mutations, precore stop codon G1896A mutation, rtM204I mutation, rtM204V mutation, rtL180M mutation were described previously [10,26]. To identify pre-S internal deletion mutations, the pre-S region flanked by P1, 5'-GCGGGTCACCATATTCTTGGGAAC-3' (nt. 2821 to 2844, sense) and P2, 5'-GAGCAGGGGTCCTAGGAATC-3' (nt. 196 to 177, antisense) was amplified by PCR. The expected size was 596 bp in length. These PCR products were subjected to Southern blot analysis to identify positively hybridized bands < 500 bp in length. PCR products 500 to 596 bp in length were gel-purified and subjected to direct sequencing to identify pre-S deletion mutants with small deleted regions (< 100 bp in length). If a mixture of wild type and deletion mutants was found by direct sequencing, the gel-purified PCR product was cloned to pCR2.1-TOPO vector (Invitrogen, Carlsbad, CA) and 10-15 clones were sequenced to identify the deletion mutants.

**Figure 1:**
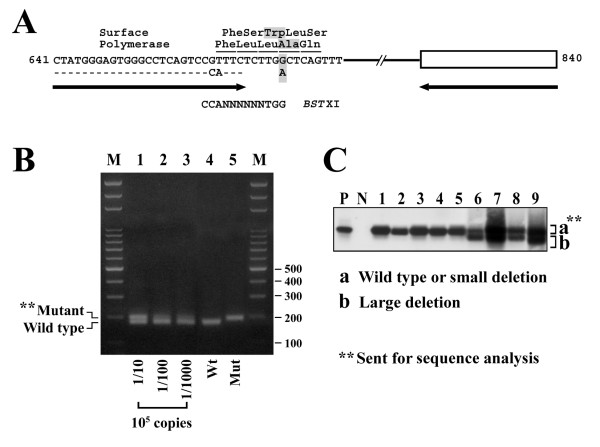
**Methods to detect rtA181T mutations and pre-S internal deletion mutations**. (A) Rationale of the amplification created restriction enzyme method to assist the detection of a small percentage of the rtA181T mutant. Partial amino acid sequences of the surface and polymerase genes were shown. Arrow (left), a mismatched primer with two nucleotides (CA) mismatches; Arrow (right), a matched antisense primer. A *BST *XI site was created if the amplicon was wild type. (B) Digestion of the PCR products with *BST *XI separated the wild type amplicon from the mixture. The undigested (mutant) DNA was gel-purified and submitted for sequence verification. In a 10^5 ^copies of mixture, this method could detect 1/1000 (10^2 ^copies) of mutant. (C) Detection of the pre-S internal deletion mutants. The PCR product was analyzed by Southern blot to detect large deletion (b). The pre-S sequence with or without small deletions (a) was gel-purified and sent for sequence analysis.

Previous studies indicated that the rtA181T mutant usually coexisted with wild type or mutant type viruses as a mixture [10,13]. As such, we developed an amplification created restriction enzyme site (ACRES) method to detect a small percentage of the rtA181T mutant. As shown in Figure 1A and 1B, a partially mismatched primer was designed. A1, 5'-CTATGGGAGTGGGCCTCAGTCCCATTC-3' (nt. 641-667, sense; mismatched nucleotides underlined). This primer and A2, 5'-GTTTAAATGTATACCCAGAGAC-3' (nt. 840-819; antisense) were used for PCR. Owing to the mismatched nucleotides, a *BST *X1 site was created in the PCR product (200 bp) if wild type sequence (but not mutant type) was amplified. The PCR product was then digested with *BST *X1. The wild type amplicon could be digested to generate a band of 170 bp in length, leaving the mutant amplicon undigested. The mutant amplicon was then gel-purified and sent for direct sequencing for confirmation. In a PCR reaction, this method was capable of detecting 100 copies of mutant when mixed with 10^3 ^to 10^7 ^copies of wild type HBV-DNA (≦ 1% of mutant could be detected) and 10 copies of mutant when mixed with 100 copies of wild type HBV-DNA (10% of mutant can be detected under low copy numbers of HBV-DNA).

### Statistical analysis

Parametrical data were expressed in mean ± standard deviation (SD) when assumed normal distribution but in median (range) when they were not. Student-t test or Mann-Whitney test was used when appropriate. Fisher's exact test was used for dichotomous data. Logistic regression analysis was performed to evaluate the association of the clinical and virological variables with occurrence of the rtA181T mutant. The Kaplan-Meier method was used to estimate the accumulative incidence of HCC and the log-rank test was used to compare between groups. The Cox proportional hazard model was used to identify independent clinical and virological factors associated with HCC occurrence. Alpha error was adjusted according to the method of Bonferroni correction in multiple comparisons. Statistical analysis was conducted by the use of SPSS (version 13.0).

## Results

### Genotypic characterization

Virological assays were performed for all 123 chronic hepatitis B patients developing LAM-resistance. The difference in the prevalence of BCP A1762T/G1764A mutation between genotype B and C HBV was statistically significant (P < 0.001; Table 1). In this study, 3 patients had small (< 100 bp) pre-S internal deletions, 27 had large (> 100 bp) pre-S internal deletions, and 2 had both kinds of pre-S deletions (Figure 1C). These 32 patients were grouped for analysis. The rtA181T mutation was detected in 10 patients. In these patients, the rtA181T mutation was accompanied by the sW172* mutation in the S reading frame. Additionally, small and large pre-S internal deletions were observed in 1 and 5 patients, respectively. No significant association was found between different genotypes and other clinical and virological parameters.

**Table 1:** Clinical and virological characteristics in LAM-resistant chronic hepatitis B patients

Characteristics	Genotype B	Genotype C	P Value^a^
Number of patients	84	39	
Male (%)	69 (82.1%)	32 (82.1%)	0.999
Age (years)	46.0 ± 10.7	48.3 ± 10.2	0.276
rtM204V (%)	31 (36.9%)	13 (33.3%)	0.840
rtM204I (%)	40 (47.6%)	22 (56.4%)	0.439
rtM204V+rtM204I (%)	13 (15.5%)	4 (10.3%)	0.578
rtL180M (%)	22 (26.2%)	12 (30.8%)	0.666
rtA181T (%)	6 (7.1%)	4 (10.3%)	0.724
HBeAg (%)	40 (47.6%)	25 (64.1%)	0.120
AST (IU/L)	146.5 ± 211.8	140.3 ± 222.7	0.886
ALT (IU/L)	223.1 ± 341.3	181.1 ± 287.6	0.506
Alpha-fetoprotein (ng/dL)	4 (0-15)	3 (0-18)	0.987
HBV-DNA (10^6 ^copies/mL)	14.1 (0.001-16063)	10.0 (0.0015-1203)	0.493
BCP 1762/1764 mutations (%)	28 (33.3%)	31 (79.5%)	< 0.001
Precore stop codon mutation (%)	45 (53.6%)	11 (28.2%)	0.0112
Pre-S internal deletions (%)	17 (20.2%)	15 (38.5%)	0.0461
Cirrhosis	26 (31.0%)	14 (35.9%)	0.680
Duration of LAM treatment before inclusion (months)	13 (6-55)	14 (6-48)	0.794

Values were given as either mean ± SD, median (range) or number (%).

^a^According to the method of Bonferroni correction for multiple comparisons, P < 0.0029 was considered statistically significant.

Of the 123 LAM-resistant patients, 58 (47.2%) subsequently received ADV (switch or add-on) rescue therapy, 20 (16.3%) received ETV (switch) rescue therapy, and 24 (19.5%) received ETV and ADV therapy sequentially secondary to the emergence of additional drug resistant mutants during their treatment courses. The remaining 21 (17.1%) patients continued to receive LAM monotherapy either because they had only mild hepatitis activities or because of barriers to access of rescue therapy.

### Clinical and virological factors associated with HCC occurrence

**Figure 2:**
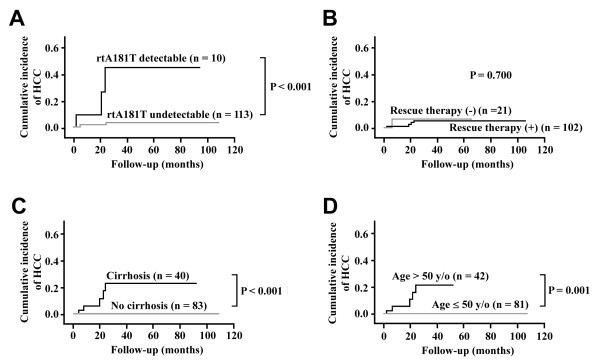
**Factors associated with occurrence of HCC**. The cumulative incidence of HCC was depicted according to the presence of the rtA181T mutation (A), use of rescue therapy (B), the presence of liver cirrhosis (C), and age > 50 years (D).

Patients were followed for a mean period of 26.2 ± 16.4 months (range 2 to 108 months). During this period, 5 patients developed HCC. Kaplan-Meier analysis was performed to estimate the association between HCC occurrence and each of the clinical and virological variables (Table 2). It was found that only age > 50 years (P = 0.001), the presence of rtA181T mutation (P < 0.001), and liver cirrhosis (P < 0.001) were significantly associated with occurrence of HCC (Figure 2). No statistically significant difference was found between those with and without pre-S internal deletions. Multivariate Cox proportional hazards regression analysis using the forward stepwise model showed rtA181T to be the only variable that remained in the equation as an independent predictor (Hazard Ratio, 21.443; 95% CI, 3.556 to 129.302; P = 0.001).

**Table 2:** Kaplan-Meier analysis for the association between clinical features and occurrence of HCC or severe consequences of liver diseases in LAM-resistant chronic hepatitis B patients

Clinical and virological factors	No. of patients	HCC		Severe liver consequences	
			
		Cumulative incidence	P (Log Rank test)^a^	Cumulative incidence	P (Log Rank test)^a^
Sex					
Male	101	5 (5.0%)	0.339	7 (6.9%)	0.775
Female	22	0 (0%)		1 (4.5%)	
Age (years)					
≦ 50	81	0 (0%)	0.001^b^	1 (1.2%)	0.001^b^
> 50	42	5 (11.9%)		7 (16.7%)	
rtM204V					
Absence	62	2 (3.2%)	0.589	2 (3.2%)	0.131
Presence	61	3 (4.9%)		6 (9.8%)	
rtM204I					
Absence	44	2 (4.5%)	0.857	3 (6.8%)	0.917
Presence	79	3 (3.8%)		5 (6.3%)	
rtM204V+I					
Absence	106	4(3.8%)	0.568	5 (4.7%)	0.033
Presence	17	1 (5.9%)		3 (17.6%)	
rtL180M					
Absence	89	3 (3.4%)	0.591	6 (6.7%)	0.830
Presence	34	2 (5.9%)		2 (5.9%)	
rtA181T					
Absence	113	2 (1.8%)	< 0.001^b^	4 (3.5%)	< 0.001^b^
Presence	10	3 (30.0%)		4 (40.0%)	
HBeAg					
Absence	58	5 (8.6%)	0.012	7 (12.1%)	0.016
Presence	65	0 (0%)		1 (1.5%)	
HBV-DNA (×10^6 ^copies/mL)					
≦ 15	69	2 (2.9%)	0.325	4 (5.8%)	0.570
> 15	54	3 (5.6%)		4 (7.4%)	
AST					
≦ 2 ×UNL	67	2 (3.0%)	0.337	2 (3.0%)	0.050
> 2 ×UNL	56	3 (5.4%)		6 (10.7%)	
ALT					
≦ 2 ×UNL	59	2 (3.4%)	0.722	3 (5.1%)	0.450
> 2 ×UNL	64	3 (4.7%)		5 (7.8%)	
BCP mutation					
Absence	64	0 (0%)	0.012	0 (0%)	0.002^b^
Presence	59	5 (8.5%)		7 (11.9%)	
Precore stop codon mutation					
Absence	67	1 (1.5%)	0.094	3 (4.5%)	0.285
Presence	56	4 (7.1%)		5 (8.9%)	
Genotype C					
Absence	84	3 (3.6%)	0.631	4 (4.8%)	0.234
Presence	39	2 (5.1%)		4 (10.3%)	
Pre-S internal deletions					
Absence	91	4 (4.4%)	0.749	6 (6.6%)	0.941
Presence	32	1 (3.1%)		2 (6.3%)	
Duration of LAM treatment on entry (months)					
≦ 12	80	2 (2.5%)	0.798	4 (5.0%)	0.361
> 12	43	3 (7.0%)		4 (9.3%)	
Subsequent ADV treatment					
No	41	1 (2.4%)	0.688	2 (4.9%)	0.774
Yes	82	4 (4.9%)		6 (7.3%)	
Subsequent ETV treatment					
No	79	4 (5.1%)	0.422	0 (0%)	0.153
Yes	44	1 (2.3%)		8 (18.2%)	
Subsequent ETV and/or ADV treatment					
No	21	1 (4.8%)	0.284	1 (4.8%)	0.181
Yes	102	4 (3.9%)		7 (6.9%)	
Total duration of antiviral therapy (months)					
≦ 40	73	1 (1.4%)	0.157	3 (4.1%)	0.343
> 40	50	4 (8.0%)		5 (10%)	
Cirrhosis					
No	83	0 (0%)	< 0.001^b^	0 (0%)	< 0.001^b^
Yes	40	5 (12.5%)		8 (20.0%)	

^a^Kaplan-Meier analysis; ^b^According to the method of Bonferroni correction for multiple comparisons, P < 0.0024 was considered statistically significant.

**Figure 3:**
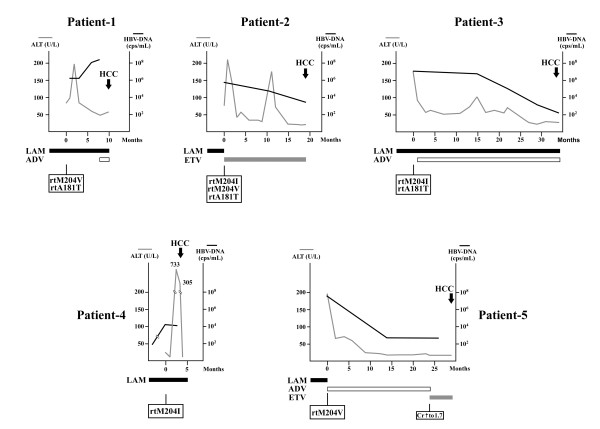
**Clinical courses of 5 HCC patients from entry to the study to development of HCC**. The changes of ALT level (gray lines) and HBV-DNA level (black line) were depicted. Arrow, time-point of HCC diagnosis; Solid bar, duration of LAM used; Shaded bar, duration of ETV used; Empty bar, duration of ADV used.

Among the three signficicant factors, it was found that all 5 HCC patients belonged to the older age (> 50 years of age) and cirrhosis groups (Figure 2). In this study, 23 patients (18.7%) met both clinical criteria. In this subset of patients, the cumulative incidence of HCC was 21.7% in a mean follow-up period of 22.0 ± 13.0 months. Clinical course of the 5 HCC patients were reviewed (Figure 3). It was discovered that rescue antiviral therapy was successful in 3 of the 5 LAM-resistant patients (patient-2, 3, and 5). In patient 1 and 4, rescue therapy was delayed because of barriers to access. In patient 1, the liver biochemistry data improved after initial hepatitis exacerbation. Notably, in the three patients with rtA181T mutants (patients-1 to 3), a relatively high level of HBV-DNA (> 10^4 ^copies/mL) persisted for at least 10 months.

### Clinical and virological factors associated with severe liver consequences

Three patients developed acute liver failure during the follow-up periods. Of these 3 patients, 2 had severe hepatitis exacerbations at 2 and 2.5 months respectively after detection of LAM-resistance. These two patients died of hepatic decompensation. The third patient developed liver failure 3 months after detection of LAM-resistance, but he underwent liver transplantation within 1 m. Taken together, 8 patients (5 with HCC and 3 who progressed to liver failure) developed severe consequences of liver diseases. Kaplan-Meier analysis was performed to estimate the association between severe liver consequences and each of the clinical and virological variables (Table 2). It was found that age > 50 years (P = 0.001), the presence of rtA181T mutation (P < 0.001), BCP A1762T/G1764A mutations (P = 0.002) and liver cirrhosis (P < 0.001) were significantly associated with severe liver consequences. No statistically significant difference was found between those with and without pre-S internal deletions. Notably, no association was found between disease outcomes and HBV-DNA level or use of rescue drugs (Table 2). Multivariate Cox proportional hazards regression analysis using the forward stepwise model showed rtA181T to be the only variable that remained in the equation as an independent predictor (Hazard Ratio, 13.091; 95% CI, 3.255 to 52.653; P < 0.001).

### Factors associated with development of rtA181T mutant

Since rtA181T mutation was identified as an independent predictor for severe consequences of liver disease including HCC and liver failure, linear regression analysis was performed to identify factors associated with emergence of rtA181T mutation (Table 3). With Bonferroni correction for multiple comparisons, P < 0.0029 was considered statistically significant. It was found that only older age was significantly associated with development of rtA181T mutant (OR, 1.144; 95%CI, 1.056-1.240; P = 0.001).

**Table 3:** Univariate logistic regression analysis for the association between clinical features and emergence of rtA181T mutation in LAM-resistant patients

Clinical factors	rtA181T		OR (95%CI)	P Value
			
	Yes (n = 10)	No (n = 113)		
Sex (male)	10 (100%)	91 (80.5%)	> 10^6 ^(0-> 10^6^)	0.998
Age (years)	58.5 ± 8.3	45.7 ± 10.1	1.144 (1.056-1.240)	0.001^a^
rtM204V	2 (20.0%)	42 (37.2%)	1.582 (0.423-5.909)	0.495
rtM204I	4 (40.0%)	58 (51.3%)	2.366 (0.480-11.670)	0.290
rtM204V+I	4 (40.0%)	13 (11.5%)	5.128 (1.276-20.607)	0.021
rtL180M	3 (30.0%)	31 (27.4%)	1.134 (0.276-4.663)	0.862
HBeAg	2 (20.0%)	63 (55.8%)	0.198 (0.040-0.976)	0.047
HBV-DNA (10^6 ^copies/mL)	15 (0.0015-16063)	2.08 (0.001-4640)	1.000 (0.999-1.001)	0.688
AST (IU/L)	147.0 ± 220.2	114.1 ± 122.1	0.999 (0.996-1.003)	0.693
ALT (IU/L)	217.3 ± 335.9	124.7 ± 124.7	0.999 (0.995-1.002)	0.408
Alpha-fetoprotein (ng/dL)	3 (0-18)	4 (0-17)	0.998 (0.997-1.001)	0.876
BCP mutation	7 (70.0%)	52 (46.0%)	2.737 (0.674-11.124)	0.159
Precore stop codon mutation	4 (40.0%)	52 (46.0%)	0.782 (0.209-2.922)	0.715
Genotype C	4 (40.0%)	35 (31.0%)	1.486 (0.394-5.598)	0.559
Pre-S internal deletions	3 (30.0%)	29 (25.7%)	2.024 (0.532-7.693)	0.301
Cirrhosis	8 (80.0%)	32 (28.3%)	10.125 (2.039-50.281)	0.005
Duration of LAM treatment (months)	13 (6-55)	13.5 (6-48)	1.043 (0.990-1.099)	0.117

Values were given as either mean ± SD, median (range) or number (%).

^a^According to the method of Bonferroni correction for multiple comparisons, P < 0.0029 was considered statistically significant.

Multiple linear regression analysis using the forward stepwise method showed that age (OR, 1.126; 95% CI, 1.032 to 1.229; P = 0.008) and cirrhosis (OR, 5.671; 95% CI, 1.063 to 30.248; P = 0.042) remained in the equation as independent predictors.

## Discussion

LAM is the first approved oral antiviral agent for the treatment of chronic hepatitis B and has been therefore widely used as the first line therapeutic agent in the past decades. Despite the availability of other NAs carrying lower risk of drug resistance (such as ADV and ETV), LAM remains to be a popular anti-HBV agent in many parts of the world because of its low cost, excellent safety profile in long term use, and absence of oncogenic potential in animal studies. However, because of widespread use, an increasing number of LAM-resistant patients have emerged.

Currently, the most widely accepted strategy for rescue therapy is ADV add-on treatment [21]. The de novo development of the cross-resistant mutant, rtA181T, was about 4% in a 4-year period of combination treatment with LAM plus ADV [27]. Previous studies also suggested effective viral suppression in LAM-resistant patients by adopting ADV or ETV monotherapy. However, it was found that the risk of developing ADV or ETV drug resistance was high with this strategy [21]. Recently, it was shown that tenofovir is superior to ADV in treating LAM-resistant patients with a better viral suppression and no detectable genotypic resistance [18,19].

Despite the availability of rescue therapy, severe disease outcomes still occurred in a substantial percentage of patients. Previous studies indicated that successful viral suppression in ADV add-on therapy did not prevent HCC occurrence in patients with liver cirrhosis [22,24]. This study demonstrated that age > 50 years, cirrhosis, BCP mutation, and the presence of the rtA181T mutation, prior to initiation of rescue therapy were significantly associated with severe liver consequences. Interestingly, neither the choice of rescue drugs nor the duration of antiviral drug use seemed to affect the clinical outcomes of these patients. In fact of the 21 patients receiving LAM monotherapy, only 1 developed HCC. It is possible that rescue therapy was not initiated in these patients because they had mild hepatitis despite development of drug resistance. Notably, two important variables, HBV-DNA level and pre-S internal deletion mutants, which were frequently identified as prognostic indicators in treatment-naïve patients were not found to be associated with clinical outcomes in LAM-resistant patients. It is likely that during rescue therapy in these patients, HBV replication was suppressed to a low level, resulting in reduction in the development of the pre-S internal deletion mutations. In this view, the pre-S internal deletion mutants are unlikely to play an important role in hepatocarcinogenesis in this subset of patients.

The most striking finding in this study was that pre-existing rtA181T mutant in LAM-resistant patients was independently associated with severe liver consequences, especially occurrence of HCC. The rtA181T mutant has been shown to confer ADV-resistant [28-30]. In ADV add-on studies, it was found that rtA181T mutants could be either pre-existing in LAM-resistant patients or de novo-developed after ADV add-on therapy [27]. Furthermore, because the reading frame of HBV polymerase overlaps with that of the S gene, some resistant mutations (such as rtA181T) in the polymerase gene coexist with S gene mutations, including stop codon mutations [31]. In some patients with rtA181T mutants, a concomitant sW172* mutation was observed in the overlapping S reading frame, resulting in truncation of the pre-S/S proteins [32]. Several previous studies have reported the transactivation activities of a truncated pre-S2 mutant isolated from the chromosomal integrated HBV genome of hepatoma cells. Such mutants were capable of transactivating several promoters including those of oncogenic proteins [33]. Transactivation activity occurred only when the premature stop codons are located within a region of the S gene, named "transactivity-on-region" [34]. The premature stop codon of the S gene in the rtA181T/sW172* mutant was located near the border of the transactivity-on-region and its transactivating activity was recently confirmed [35,36]. Additionally, owing to secretory defect and a dominant negative effect on wild-type HBV virion secretion, emergence of rtA181T/sW172* largely reduced the typical extent of virological breakthrough in serum, resulting in difficulty to recognize drug resistance [32]. In this study, a hypersensitive ACRES method was used to detect small percentages of the rtA181T mutants. Of the 10 rtA181T-positive patients, the mutants constituted < 5%, 10-30% and > 90% of the viral population in 6, 2, and 2 patients, respectively. We speculated that despite a small percentage of the mutants was detected in the serum, a larger percentage could exist in the liver, owing to the secretory defect of the sW172* mutant. Presumably, intrahepatic accumulation of this mutant partially contributed to its oncogenic effect.

Finally, in our study, 23 patients had two unfavorable conditions: liver cirrhosis and older than 50 years of age. During the follow-ups, 5 of the 23 patients (21.7%) developed HCC and 7 of the 23 (30.4%) patients developed severe liver consequences. The present data indicated that in this special subgroup of patients, careful selection of antiviral agent and closely monitoring for viral mutants (including BCP and rtA181T mutations) are mandatory.

## Conclusion

By analyzing the clinical variables, virological parameters, and clinical use of rescue agents in LAM-resistant mutants, we found older age, cirrhosis, and rtA181T mutation are significantly associated with occurrence of HCC in the subsequent courses of antiviral therapy.

## Competing interests

The authors declare that they have no competing interests.

## Authors' contributions

CTY designed the study and analyzed the clinical data. CTY and TC involved in drafting the manuscript and revising it critically for important intellectual content. CWH, YCC, KHL, and MWL interpreted and analyzed the clinical and laboratory data. TCC interpreted and analyzed the pathological data. All authors have read and approved the final manuscript.

## Pre-publication history

The pre-publication history for this paper can be accessed here:

http://www.biomedcentral.com/1471-2407/11/398/prepub
